# Book Reviews

**Published:** 1908-01

**Authors:** 


### BOOK REVIEWS.

——o——

### IMMUNE SERA.

A concise exposition of our present knowledge concerning the constitution and mode of action of antitoxins, agglutinins, haemolysins, bacteriolysins, preciptins, cyttotoxins and opsomins, by Dr. C. F. Boldnan, Bacteriologist, Research Laboratory, Department of Health, New York. Price $1.50. John Wiley and Sons, Publishers, New York.

The basis for this scientific and up-to-date little book, was a monograph by Prof. Wassermann which was translated and published by Dr. Boldnan in 1904 under the title "Immune Sera." The original topics have been discussed more fully and the scope of the book widened by adding chapters on snake venoms, agglutinins, opsomins and serum sickness.

This fascinating little book is thoroughly up with all the advances and recent discoveries in this productive field, and provides one with the gist of the voluminous literature bearing on these subjects.

PHYSICAL CHEMISTRY IN THE SERVICE OF MEDICINE, seven addresses by Dr. Wolfgang Pauli, Vienna, authorized translation by Dr Martin H. Fisher; John Wiley & Sons, Publishers, New York.

This volume is composed of Dr. Pauli's addresses on the application of physical chemistry to different fields in medicine as rendered possible more particularly through the advances in the physics and chemistry of organic colloids. They are in the main summaries of the special investigations he has made in this interesting, but rather advanced subject.

---

### VEST POCKET SERIES OF ABSTRACTS OF THE STANDARD TEXT BOOKS ON MEDICINE.

---

Anatomy by McCurdy; Histology and Bacteriology by Wallgren; Chemistry by Inglis; Materia Medica by Orr; Physiology by Rhodes; and Emergencies by McCurdy. Published by the Medical Abstract Publishing Company, Pittsburg, Pa.

These excellent little books are not intended as a substitute for the text books, but as an aid to these works, bringing out and emphasizing the essential points. They contain everything on the various subjects that will be included in the final examination, either in college, or before the State Board.

Their size enables the doctor or student to carry them about at all times, so that he can study whenever he has a moment to spare, and thus thoroughly post himself upon these subjects.

---

### DISEASES OF THE NERVOUS SYSTEM.

---

Edited by Archibald Church, M.D.. Translation from "Die Deutch Klinik" under the general editorial supervision of Julius L. Salinger, M.D. With one hundred and ninety-five illustrations, and five colored plates; twelve hundred pages. D. Appleton and Co., New York.

This is an excellent composite work and although perhaps lacking some of the homogeniety of a book written by one author, this is more than compensated for by the excellence and care each contributer has taken to make his chapter as nearly perfect as possible. These contributers having been selected on account of their proficiency in particular fields of work, makes this a most valuable book for both the practitioner and the nerve specialist. The subject is presented in a systematic manner and covers thoroughly all of the important diseases of the nervous system.

Among the well known contributors are: Rothmann, Rosin, Schuster and Bernhardt of Berlin; Redlich, Frankel-Hochwast of Vienna; Quinke of Kiel; Wernicke of Breslau; Schultze of Bonn; Erb of Heideberg; and Von Leyden, Lazarus and Cassier of Berlin. This array of writers renders the work authoritative. The cuts are clear and instructive.

